# Correlation between Lymphocyte-to-Monocyte Ratio (LMR), Neutrophil-to-Lymphocyte Ratio (NLR), Platelet-to-Lymphocyte Ratio (PLR) and Extramural Vascular Invasion (EMVI) in Locally Advanced Rectal Cancer

**DOI:** 10.3390/curroncol30010043

**Published:** 2022-12-30

**Authors:** Cieszymierz Gawiński, Anna Hołdakowska, Lucjan Wyrwicz

**Affiliations:** 1 Maria Sklodowska-Curie National Research Institute of Oncology, ul. Wawelska 15, 02-034 Warsaw, Poland; 2 Department of Radiology, National Research Institute of Oncology, ul. Roentgena 5, 02-781 Warsaw, Poland; 3 Department of Oncology and Radiotherapy, National Research Institute of Oncology, ul. Wawelska 15, 02-034 Warsaw, Poland

**Keywords:** lymphocyte-to-monocyte ratio (LMR), neutrophil-to-lymphocyte ratio (NLR), platelet-to-lymphocyte ratio (PLR), extramural vascular invasion (EMVI), rectal cancer

## Abstract

Rectal cancer constitutes around one-third of all colorectal cancers. New markers are required to optimize the treatment. Extramural vascular invasion (EMVI) is a magnetic resonance imaging (MRI)-based negative prognostic marker. Lymphocyte-to-monocyte ratio (LMR), neutrophil-to-lymphocyte ratio (NLR) or platelet-to-lymphocyte ratio (PLR) are blood-based systemic inflammatory response markers with proven prognostic value in many cancers, including CRC. We hypothesized whether there is a relationship between LMR, NLR, PLR and the presence of EMVI on pre-treatment MRI in patients with locally advanced rectal cancer (LARC). We conducted a retrospective analysis of 371 patients with LARC treated in the Maria Skłodowska-Curie National Research Institute of Oncology, Warsaw, Poland between August 2016 and December 2021. One hundred eighty-four patients were found eligible for the study. A correlation between the extension of the tumour, nodal status, clinical stage of the disease and the presence of EMVI was found (*p* < 0.001). The pre-treatment level of neutrophils, platelets and carcinoembryonic antigen (CEA) was significantly higher in the EMVI-positive population (*p* = 0.041, *p* = 0.01, *p* = 0.027, respectively). There were no significant differences regarding the level of LMR, NLR and PLR between the EMVI-positive and EMVI-negative population. LMR, NLR and PLR do not differentiate patients in terms of EMVI; neither of these parameters is a good predictor of the status of EMVI in LARC.

## 1. Introduction

Colorectal cancer (CRC) is the third most common cancer and the second most common cause of cancer-related death worldwide [1] Rectal cancer, which constitutes around one-third of all CRCs, is distinct from colon cancer with different etiology, risk factors, pre-treatment staging and treatment strategies. The incidence of rectal cancer in the European Union is estimated to be as high as 125 000 per year and is expected to increase in the future [2]. Locally advanced rectal cancer (LARC) is usually defined as stage II or III disease (T3-T4, node-negative or node-positive irrespective of the extension of the tumor). Neoadjuvant radio/chemo-radiotherapy followed by surgery according to total mesorectal excision principles is deemed to be the standard of care. Treatment modalities differ substantially among countries and research centers. There is a growing need for novel markers in order to identify high-risk patients and optimize the treatment. Until recently, the Union for International Cancer Control (UICC) tumor node metastasis (TNM) staging system has been a backbone for treatment planning and main prognostic factor [3]. With the development of magnetic resonance imaging (MRI) technology, high-resolution MRI has become the standard preoperative diagnostic tool in rectal cancer [4]. Extramural vascular invasion (EMVI) is defined as an invasion of malignant cells into blood vessels (usually veins). Detection of EMVI on pre-treatment MRI is associated with an increased risk of distant metastases and reduced disease-free survival. [5]. Blood-based systemic inflammatory response (SIR) markers are other emerging biomarkers with a well-established prognostic value in many cancers. Lymphocyte-to-monocyte ratio (LMR), neutrophil-to-lymphocyte ratio (NLR) and platelet-to-lymphocyte ratio (PLR) are among the most investigated ones. Low LMR, high NLR and high PLR are linked to unfavorable prognosis. This pattern has been consistently confirmed in various malignancies [6,7,8]. The prognostic value of SIR markers in colorectal cancer has been well documented in both locally advanced and metastatic stages [9,10,11,12,13,14,15]. We hypothesized whether there is a correlation between radiological and blood-based prognostic markers allowing for SIR markers to act as surrogates of the status of EMVI. In this study, we investigated the relationship between LMR, NLR, PLR and the presence of EMVI on pre-treatment MRI in LARC patients.

## 2. Materials and Methods

**Figure 1:**
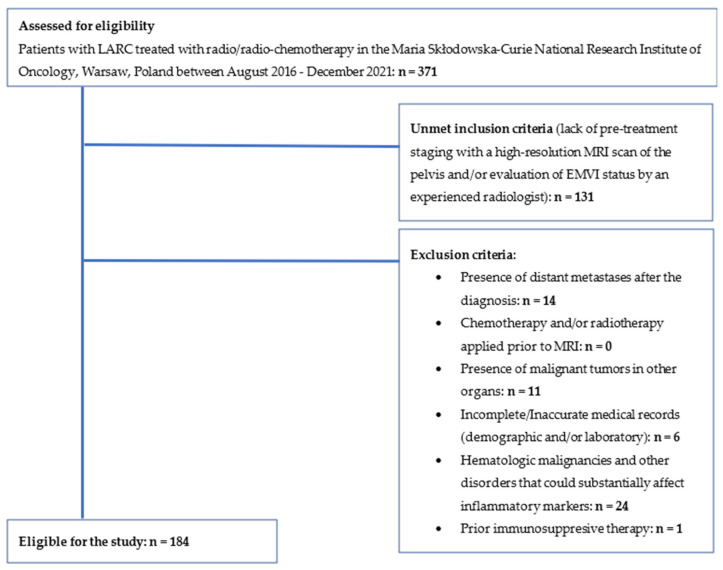
Eligibility for the study. LARC, locally advanced rectal cancer; MRI, magnetic resonance imaging; EMVI, extramural vascular invasion.

A retrospective analysis of a database of 371 patients with LARC treated in the Maria Skłodowska-Curie National Research Institute of Oncology, Warsaw, Poland between August 2016 and December 2021 was performed. The inclusion criteria were as follows: (1) patients were diagnosed with primary locally advanced rectal cancer (T3-T4, N0-N2, M0) confirmed by histopathology. TNM stage was assessed according to the American Joint Committee on Cancer TNM staging standard, 8th edition; (2) pre-treatment staging with a high-resolution MRI scan of the pelvis and evaluation of EMVI status by an experienced radiologist was performed; (3) performance status of the patients was ECOG 0–2, patients were qualified to receive radio/chemo-radiotherapy by multidisciplinary team; (4) clinical records including demographic and laboratory data were available and complete. The exclusion criteria were: (1) presence of distant metastases after the diagnosis; (2) chemotherapy and/or radiotherapy applied prior to MRI; (3) presence of malignant tumors in other organs; (4) presence of hematologic malignancies and disorders that could substantially affect inflammatory markers; (5) prior immunosuppressive therapy. One hundred eighty-seven patients were excluded from the study due to unmet inclusion criteria or the presence of exclusion criteria. One hundred eighty-four were found eligible for the study as shown in Figure 1.

Blood examination of each patient has been analyzed and level of LMR, NLR and PLR has been calculated as presented in Table 1. The pre-treatment level of carcinoembryonic antigen (CEA) and carbohydrate antigen 19-9 (CA 19-9) was collected when available (164/184 cases). The median time between blood examination and MRI was 8 days (range 0–43 days). The differential white blood cell count was analyzed using the Sysmex XN-550 hematology analyzer following the manufacturer protocol. All the patients received neoadjuvant radio/chemo-radiotherapy according to the multidisciplinary team decision based on the stage of the disease. The surgery was performed on 149 patients. Post-surgical pathological results were collected and analyzed.

### 2.1. MRI Acquisition

The standard pelvic MRI was carried out using a 1.5 T-3T system, as routinely used for the clinical staging of patients with rectal cancer (according to the European Society of Gastrointestinal and Abdominal Radiology). The examination was performed with the use of a phased-array surface coil. Patients received intravenous spasmolytic; they did not receive a bowel preparation. According to the protocol two-dimensional (2D) FSE T2-weighted sequences without fat suppression, with a small field of view and a section thickness less than 3 mm (high-resolution protocol) and a diffusion-weighted sequence (including at least a high *b*-value of ≥800) have been performed. Transverse and coronal sequences were angulated perpendicular and parallel to the rectal tumor axis, respectively.

### 2.2. Assessment of Status of EMVI

**Figure 2:**
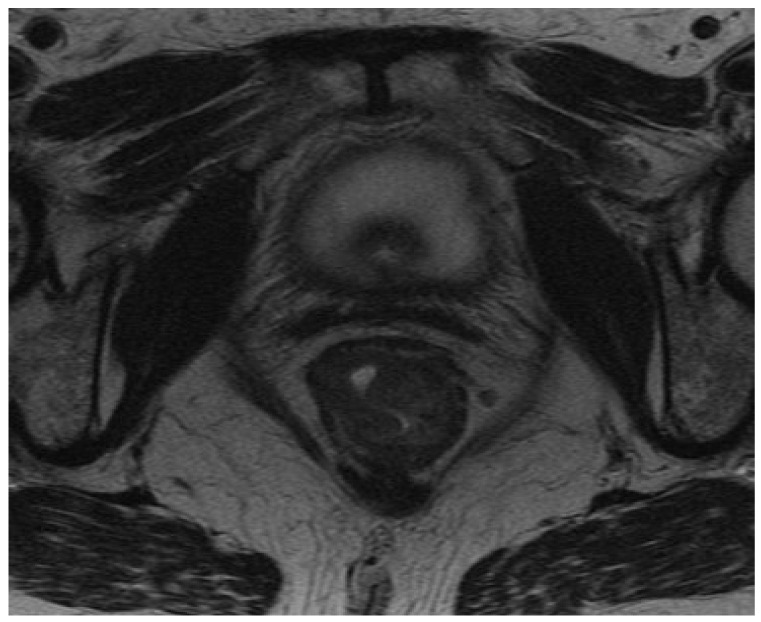
EMVI-negative rectal cancer.

**Figure 3:**
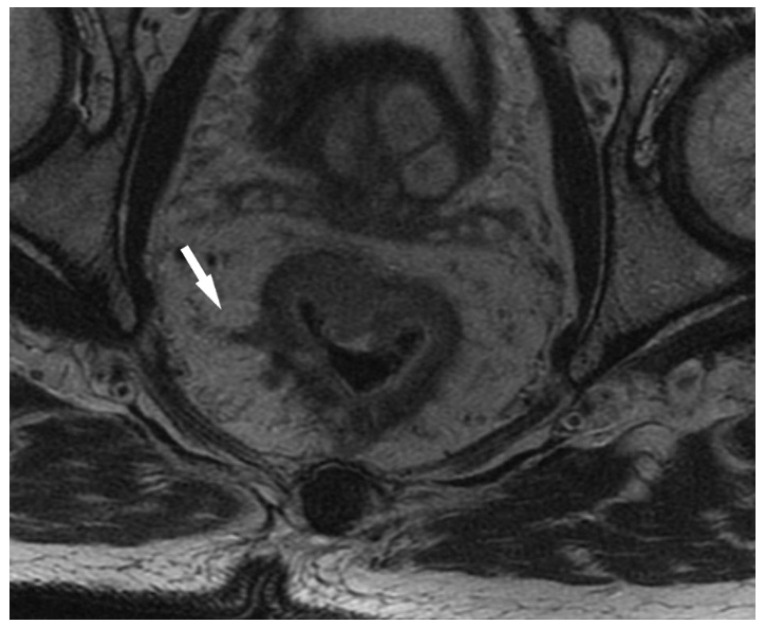
EMVI-positive rectal cancer. Axial T2-weighted MRI scan showing EMVI (white arrow).

EMVI, defined as the extension of tumor within the vessels of the mesorectum, was identified by the high-resolution MRI-based radiological features such as the vessel wall irregularity, focal enlargement, and/or signal intensity of the tumor within the vessel. To detect EMVI and minimize the risk of interobserver variability each MRI scan was reviewed independently by two radiologists with at least ten years of experience in pelvic MRI assessment. Equivocal cases were jointly discussed among experienced team of radiologists and consensus decision on the status of EMVI was made. EMVI was reported as positive/negative. Representative images of EMVI-negative and EMVI-positive rectal cancers are presented in Figure 2 and Figure 3.

### 2.3. Statistical Analysis

Analyses were conducted in statistical software R, version 4.2.2 (The R Foundation for Statistical Computing c/o Institute for Statistics and Mathematics Wirtschaftsuniversit¨at Wien Welthandelsplatz 1 1020 Vienna, Austria). The level of significance was equal to *α* = 0.05. Dependencies between groups and other qualitative variables were analysed with chi-square test or Fisher’s exact test. Quantitative variables were compared between groups using Mann–Whitney’s test. Normality of distributions was analysed with Shapiro–Wilk’s test. ROC (Receiver Operating Characteristic) analyses were performed to check whether the level of LMR, NLR and PLR differentiates patients in terms of EMVI—sensitivity, specificity and area under curve (AUC with 95% confidence intervals) were calculated, as well as optimal cut-off point for mentioned variables (based on Youden’s criterium). Kendall’s tau-b coefficient was used to assess the correlation between selected variables.

### 2.4. Ethical Considerations

The study conformed to the provisions of the Declaration of Helsinki and was approved by the ethics committee of Maria Skłodowska-Curie National Research Institute of Oncology in Warsaw.

## 3. Results

The characteristics of patients is presented in Table 1. One hundred eleven males and seventy-three females were included in the study. The mean age was 66 years old (range, 36–87 years old). The distribution of cancer stages was as follows: stage II-IIIA—84 patients (47.2%); stage IIIB—65 (36.5%) patients and IIIC—29 (16.3%) patients. Seventy-eight patients (42.4%) were EMVI-positive and one hundred six patients (57.6%) were EMVI-negative. The mean and median values of lymphocytes, monocytes, neutrophils and thrombocytes counts are presented in Table 2. The median values of LMR, NLR and PLR were 2.87, 2.62 and 153.11, respectively. The median value of CEA was 3.88 ng/mL and CA 19-9 10.04 U/mL.

There was a greater proportion of patients with T2-T3 tumors in the EMVI-negative than in the EMVI-positive group (91% vs. 66%; *p* < 0.001). In the EMVI-negative group there was 53% of patients without nodal involvement and 10% of patients with nodal status N2. In the EMVI-positive group those proportions were, respectively, 26% and 35%—presenting a statistically significant dependency (*p* < 0.001). A greater proportion of patients with stage II-IIIA and lower proportion of patients with stage IIIC were found in the EMVI-negative group than in the EMVI-positive group (58% vs. 33% for stage II-IIIA and 5% vs. 32% for stage IIIC; *p* < 0.001). The level of neutrophils, platelets and CEA was significantly lower in the EMVI-negative group than in the EMVI-positive group (*p* = 0.041 for neutrocytes, *p* < 0.001 for platelets, and *p* = 0.027 for CEA), Table 3.

All the patients received neoadjuvant radiotherapy or chemo-radiotherapy based on multidisciplinary team decisions. A greater proportion of patients with pathological stage I or II and a smaller proportion of patients with pathological stage III could be observed among EMVI-negative patients than among EMVI-positive (27% vs. 13% for stage I, 36% vs. 25% for stage II, and 24% vs. 54% for stage III; *p* = 0.002). Among EMVI- negative group there was a greater proportion of patients with pN 0 than among EMVI-positive group (75% vs. 45%) and a smaller proportion of patients with pN 1 (17% vs. 37%) or pN 2 (8% vs. 18%); *p* < 0.001. No significant correlation was observed between pT and EMVI (*p* = 0.078).

No significant correlation between pCR and the status of EMVI was detected (8.2% in EMVI-positive and 13% in EMVI-negative group; *p* = 0.350), Table 4.

Patients were assigned to groups based on the change between the MRI-based clinical TNM-staging and the post-surgical pathological TNM-staging depending on the pre-treatment status of EMVI. No significant dependency was detected between EMVI and the change of the stage, T or N status (*p* > 0.050 for all variables), Supplementary Table S1.

**Figure 4:**
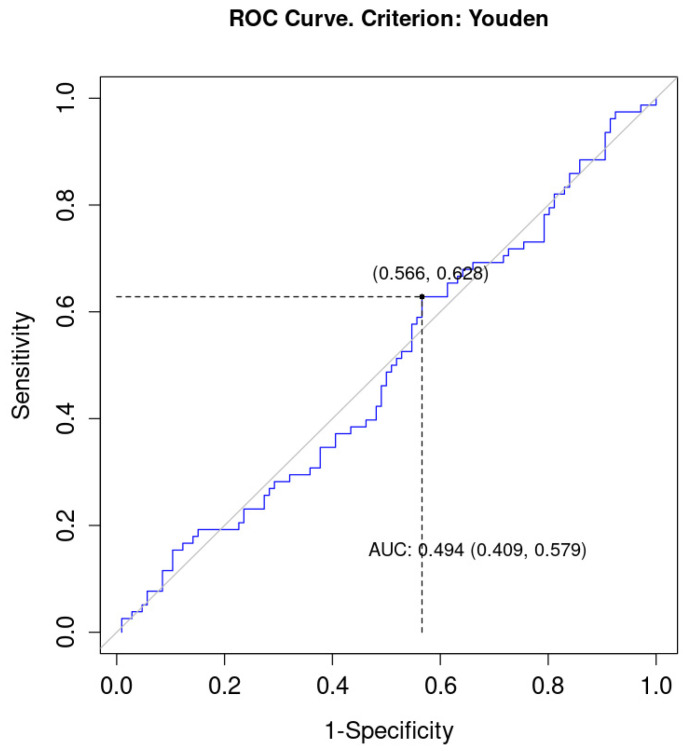
ROC (Receiver Operating Characteristic) curve for LMR as a discriminator of EMVI. AUC, area under curve.

The optimal cut-off point for level of LMR was 2.62 (sensitivity = 0.63; 1-specificity = 0.57). The AUC (area under curve) equalled 0.49 (95% CI = 0.41; 0.58), which meant that LMR level was not a good predictor of the status of EMVI, Figure 4.

**Figure 5:**
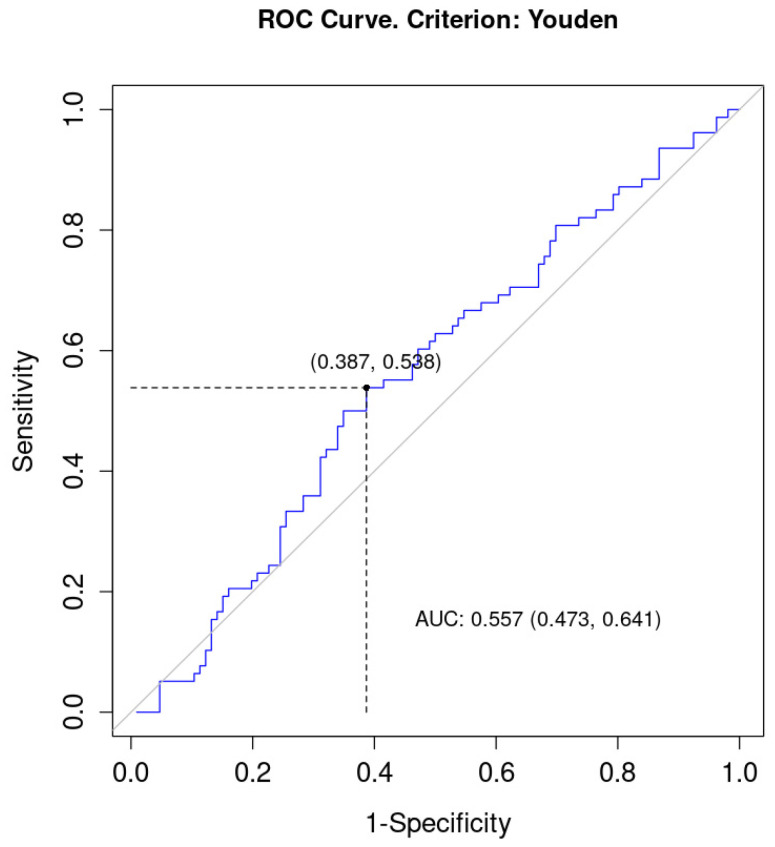
ROC (Receiver Operating Characteristic) curve for NLR as a discriminator of EMVI. AUC, area under curve.

The AUC for NLR was 0.56 (95% CI = 0.47; 0.64) which poorly differentiated patients in terms of the status of EMVI. Both sensitivity and 1-specificity were low (0.54 and 0.39, respectively). The optimal cut-off point for NLR was 2.78, Figure 5.

**Figure 6:**
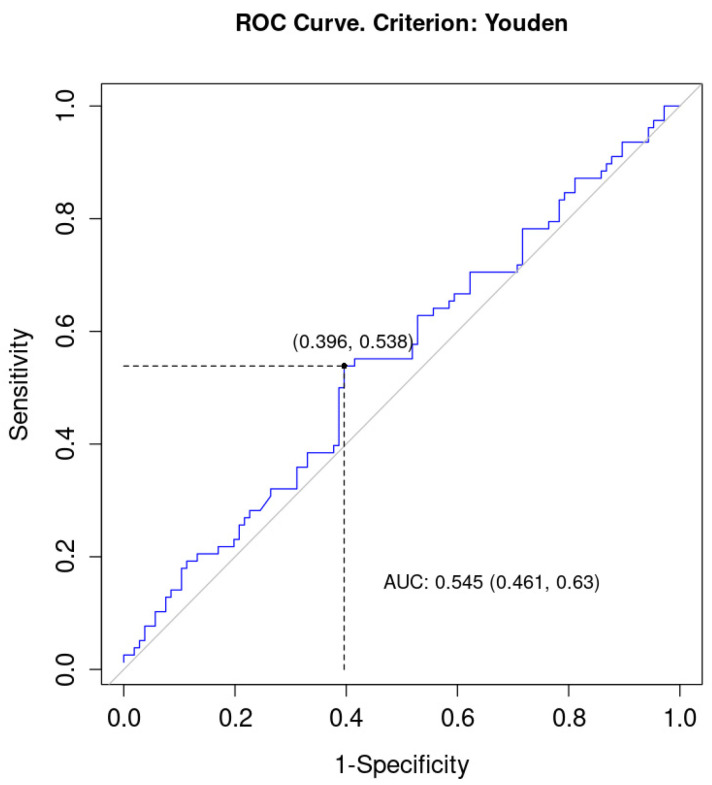
ROC (Receiver Operating Characteristic) curve for PLR as a discriminator of EMVI. AUC, area under curve.

The AUC for PLR was also higher than 0.50 and equaled 0.55 (95% CI = 0.46; 0.63), which meant that PLR was a weak discriminator of the status of EMVI. The sensitivity for this variable was 0.54, and 1-specificity equaled 0.40. The optimal cut-off point in this model was 162.71, Figure 6.

In all the analysed groups of patients according to the level of LMR, NLR and PLR there was a significant correlation between the status of EMVI and the stage of the disease.

In EMVI-positive population there were more stages IIIC and fewer stages II-IIIA than in EMVI-negative population independently from the median value of LMR, NLR and PLR (*p* < 0.050 for all analyses), Table 5.

No significant dependency was observed between pre-treatment level of CEA, CA19-9 and: LMR, NLR, PLR level (*p* > 0.050 for all correlation analyses), Table 6.

## 4. Discussion

The goal of the study was to investigate the relation between the pre-treatment MRI-based status of EMVI and SIR markers: LMR, NLR and PLR. As far as we know, our study is the first to evaluate directly such correlations. A statistically significant association between the status of EMVI and the extension of the tumor, nodal status and the clinical stage of the disease has been observed in our study. Correlation between EMVI and the stage of the disease was observed among patients with both high and low levels of LMR, NLR and PLR. A correlation between pre-treatment levels of neutrophils, thrombocytes, carcinoembryonic antigen and the status of EMVI was found (*p* = 0.041; *p* = 0.001 and *p* = 0.027, respectively). In an analysis of correlation between pre-treatment status of EMVI and pathological TNM staging there was a significant correlation between EMVI and the pathological stage (*p* = 0.002) and the pN status (*p* < 0.001). There was no significant difference in the frequency of complete pathological response in EMVI-positive and EMVI-negative patients (*p* = 0.350). The status of EMVI on pre-treatment MRI had no statistically significant impact on the change of the stage of the disease between MRI-based and post-surgical pathological TNM staging.

In accordance with our results, the relation between MRI-detected EMVI-positive rectal cancers and positive nodal status and advanced T-stage has been demonstrated in several studies [16,17,18]. In spite of significant correlation between level of neutrophils, platelets and EMVI, no such phenomenon between EMVI and LMR, NLR or PLR was detected. The available data on relations between SIR markers and EMVI in the literature is scarce. In a study by Li et al., no statistically significant association between EMVI and NLR and PLR was found in locally advanced rectal cancer patients [19]. Pine et al. investigated the relationship between NLR and the tumor characteristics and local lymphocytic response to tumor. NLR was associated with more advanced, aggressive tumor biology, lymph node metastases and EMVI (*p* = 0.07). However, the status of EMVI in the study was assessed pathologically (pEMVI), the investigated population was not confined to rectal cancer patients but included colon cancers as well and the cut-off value of NLR was relatively high (5.0 compared to 2.86 in our study) [20]. The data on correlation between EMVI and tumor markers (CEA, CA19-9) are contradictory [21,22]. No reliable information on the relation between thrombocytosis and neutrophilia and the status of EMVI has been found in the literature. EMVI has been proved to be a reliable marker of worse survival and disease recurrence [23,24]. Traditionally, EMVI was detected on pathological analysis of the resected specimens (pEMVI); however, it was demonstrated to lead to substantial under-reporting. In historical studies the incidence of pEMVI ranged from 9% to 90% as a result of an inconsistency in pathological definition and often inability to distinguish lymphatic invasion from venous invasion. In recent years, it has been proved that EMVI may be identified by high-resolution MRI at least as or more accurately compared to routine pathological analysis [25,26].

In rectal cancers EMVI has become a standard of care in pre-operative radiological assessment. In most reports it is estimated to be present in around 35% of these tumors varying from 20% to over 50% [27,28]. The role of (neo)adjuvant chemotherapy in LARC is uncertain. There is no high-quality evidence for its benefit, but it is commonly used based on data extrapolated from trials of colon cancers.

However, there is a significant variability in practice as to which treatment modalities should be offered for this group of patients. EMVI is increasingly used as one of the main factors helping to identify patients who require more intense peri-operative treatment. Chand et al. demonstrated that patients with EMVI-positive stage II tumors have a similarly increased risk of developing metastases as EMVI-negative stage III patients [29]. Meta-analysis by Siddiqui et al. revealed a significantly increased risk of both synchronous and metachronous metastases in patients with MRI-based EMVI. [28]. EMVI’s role is not limited to rectum—it’s been shown to be a strong predictor of worse oncological outcomes in stage II–III colon cancer patients; studies show promising data concerning its possible prognostic value in esophageal and gastric cancers (via computed tomography EMVI) as well [30,31,32].

LMR, NLR and PLR are novel blood-based biomarkers with strong prognostic value in various malignancies. Each of these parameters take advantage of the specific role and impact of lymphocytes, monocytes, neutrophils and platelets on immunological system. Lymphocytes through cytotoxic activity and production of anti-tumor cytokines play a key role in suppressing cancer’s proliferation and spread [33]. On the contrary, high count of monocytes, neutrocytes and platelets contribute to cancer initiation, angiogenesis, tumor progression and metastatic activity [34,35,36,37,38]. These properties have led to the introduction of lymphocyte-to-monocyte, neutrophil-to-lymphocyte and platelet-to-lymphocyte ratios with strong prognostic value. In LARC low LMR and high NLR and PLR are unfavorable prognostic factors and therefore potential biomarkers which may be used to identify high-risk patients requiring more intense treatment [10,39,40].

EMVI has been well established with SIR markers as an emerging status as prognostic factors in rectal cancer. We hypothesized that combining these parameters may enhance their prognostic value and allow for more accurate pre-treatment assessment of patients (e.g., as a part of risk-scoring models) in the future. The correlation between EMVI and SIR markers could allow to utilize cheap and easy blood-based markers as surrogates of the status of EMVI in situations when MRI is contraindicated or where the accessibility of pre-treatment MRI is still low. The main limitation of our study is a high interobserver variability in the assessment of the status of EMVI [41]. Despite the independent evaluation of each MRI scan by at least two experienced radiologists, its impact on the results is possible.

Despite the fact that no correlation between LMR, NLR, PLR and the status of EMVI was found; interesting dependencies with the clinicopathological features and pre-treatment level of neutrophils, platelets and CEA were demonstrated.

We believe our research is novel and the subject of correlations between radiological and hematological markers in rectal cancer is almost absent in the literature. We hope this study will attract more attention to the topic and encourage further investigation.

## 5. Conclusions

The presence of EMVI on pre-treatment MRI is one of the most important negative prognostic factors in rectal cancer. LMR, NLR and PLR are emerging blood-based markers with powerful prognostic value in CRC. We conducted novel research investigating the relationship between these parameters in locally advanced rectal cancer. LMR, NLR and PLR do not differentiate patients in terms of EMVI; none of these parameters seem to be a good predictor of the status of EMVI. However, the status of EMVI has been well correlated with many clinicopathological features and hematological markers. Further investigations on the possibility of utilizing these correlations in clinical practice are needed.

## Figures and Tables

**Table 1:** Calculation of LMR, NLR and PLR.

Formulas:
LMR—absolute lymphocyte count (10^9^/L)/absolute monocyte count (10^9^/L)
NLR—absolute neutrophil count (10^9^/L)/absolute lymphocyte count (10^9^/L)
PLR—absolute platelet count (10^9^/L)/absolute lymphocyte count (10^9^/L)

LARC, locally advanced rectal cancer; MRI, magnetic resonance imaging; EMVI, extramural vascular invasion.

**Table 2:** Characteristics of all patients.

Characteristics	Value
N	
EMVI +, n (%)	78 (42.4)
EMVI −, n (%)	106 (57.6)
Age (years), M ± SD, range (years)/ Me (Q1; Q3)	65.55 ± 10.83, 36–87/65.69 (58.97; 72.32)
Sex (female), n (%)	73 (39.9)
Tumor, n (%)	
T2-T3	146 (80.7)
T4	35 (19.3)
Lymph nodes, n (%)	
N0	68 (41.2)
N1	63 (38.2)
N2	34 (26.0)
Stage, n (%)	
II-III A	84 (47.2)
III B	65 (36.5)
III C	29 (16.3)
ALC (10^9^/L), M ± SD	1.77 (1.38; 2.24)
AMC (10^9^/L), Me (Q1; Q3)	0.60 (0.50; 0.76)
ANC (10^9^/L), Me (Q1; Q3)	4.77 (3.77; 5.99)
Platelets (10^9^/L), Me (Q1; Q3)	274.50 (230.75; 337.25)
LMR, Me (Q1; Q3)	2.87 (2.25; 3.81)
NLR, Me (Q1; Q3)	2.62 (2.09; 3.42)
PLR, Me (Q1; Q3)	153.11 (117.98; 204.10)
CEA (ng/mL), Me (Q1; Q3)	3.88 (2.33; 9.06)
CA19-9 (U/mL), Me (Q1; Q3)	10.04 (4.64; 17.73)

EMVI, extramural vascular invasion; ALC, absolute lymphocyte count; AMC, absolute monocyte count; ANC, absolute neutrophil count; LMR, lymphocyte-to-monocyte ratio; NLR, neutrophil-to-monocyte ratio; PLR, platelet-to-lymphocyte ratio; CEA, carcinoembryonic antigen; CA19-9, carbohydrate antigen 19-9; M, mean; SD, standard deviation; Q, quartile; Me, median.

**Table 3:** Comparison of characteristics between EMVI groups.

Characteristics	EMVI −	EMVI +	*p*
Age (years), Me (Q1; Q3)	65.90 (58.75; 74.00)	64.90 (59.48; 71.76)	0.667
Sex (female), n (%)	43 (41.0)	30 (38.5)	0.851^2^
Tumor, n (%)			
T2-T3	96 (91.4)	50 (65.8)	**<0.001^2^**
T4	9 (8.6)	26 (34.2)	
Lymph nodes, n (%)			
N0	49 (52.7)	19 (26.4)	**<0.001^2^**
N1	35 (37.6)	28 (38.9)	
N2	9 (9.7)	25 (34.7)	
Stage, n (%)			
II-III A	59 (57.8)	25 (32.9)	**<0.001^2^**
III B	38 (37.3)	27 (35.5)	
III C	5 (4.9)	24 (31.6)	
ALC (10^9^/L), Me (Q1; Q3)	1.70 (1.33; 2.24)	1.90 (1.45; 2.26)	0.217
AMC (10^9^/L), Me (Q1; Q3)	0.58 (0.46; 0.80)	0.63 (0.53; 0.75)	0.181
ANC (10^9^/L), Me (Q1; Q3)	4.49 (3.62; 5.81)	5.10 (4.07; 6.16)	**0.041**
Platelets (10^9^/L), Me (Q1; Q3)	253.50 (217.00; 313.25)	303.00 (249.50; 361.75)	**0.001**
LMR, Me (Q1; Q3)	2.90 (2.30; 3.83)	2.86 (2.20; 3.79)	0.885
NLR, Me (Q1; Q3)	2.54 (2.05; 3.31)	2.82 (2.18; 3.43)	0.189
PLR, Me (Q1; Q3)	150.70 (115.97; 200.00)	167.19 (119.95; 218.05)	0.293
LMR above median, n (%)	53 (50.0)	38 (48.7)	0.982^2^
NLR above median, n (%)	49 (46.2)	43 (55.1)	0.296^2^
PLR above median, n (%)	49 (46.2)	43 (55.1)	0.296^2^
CEA, Me (Q1; Q3)	3.62 (2.24; 6.97)	5.63 (3.07; 9.68)	**0.027**
CA19-9, Me (Q1; Q3)	9.87 (5.36; 15.76)	11.71 (2.77; 18.29)	0.741

Bold font indicates statistical significance. EMVI, extramural vascular invasion; ALC, absolute lymphocyte count; AMC, absolute monocyte count; ANC, absolute neutrophil count; LMR, lymphocyte-to-monocyte ratio; NLR, neutrophil-to-monocyte ratio; PLR, platelet-to-lymphocyte ratio; CEA, carcinoembryonic antigen; CA19-9, carbohydrate antigen 19-9; Q, quartile; Me, median. Differences in the level of quantitative variables between groups were analysed with Mann–Whitney’s U test, dependencies between qualitative variables and groups were analysed with chi-square test^2^ (with Yate’s correction for continuity for 2 × 2 tables).

**Table 4:** Correlations between EMVI and pathological staging.

Characteristics	EMVI −	EMVI +	*p*
Stage, n (%)			
pCR	12 (13.0)	5 (8.2)	0.350
I	25 (27.2)	8 (13.1)	**0.002**
II	33 (35.9)	15 (24.6)	
III	22 (23.9)	33 (54.1)	
pT, n (%)			
0	10 (11.2)	4 (6.7)	0.078
1 and 2	30 (33.7)	12 (20.0)	
3 and 4	49 (55.1)	44 (73.3)	
pN, n (%)			
0	67 (75.3)	27 (45.0)	**<0.001**
1	15 (16.9)	22 (36.7)	
2	7 (7.9)	11 (18.3)	

Bold font indicates statistical significance. EMVI, extramural vascular invasion; pCR, pathological complete response. Dependencies between qualitative variables and groups were analysed with chi-square test.

**Table 5:** Dependency between stage of the disease and EMVI (broken down into groups by the level of LMR, NLR and PLR).

Stage, n (%)	EMVI −	EMVI +	*p*
LMR ≤ median			
II–III A	29 (58.0)	15 (38.5)	**0.002**
III B	19 (38.0)	11 (28.2)	
III C	2 (4.0)	13 (33.3)	
LMR > median			
II–III A	30 (57.7)	10 (27.0)	**0.002**
III B	19 (36.5)	16 (43.2)	
III C	3 (5.8)	11 (29.7)	
NLR ≤ median			
II–III A	30 (55.6)	12 (34.3)	**0.008**
III B	21 (38.9)	13 (37.1)	
III C	3 (5.6)	10 (28.6)	
NLR > median			
II–III A	29 (60.4)	13 (31.7)	**0.001**
III B	17 (35.4)	14 (34.1)	
III C	2 (4.2)	14 (34.1)	
PLR ≤ median			
II–III A	31 (56.4)	10 (29.4)	**0.010**
III B	21 (38.2)	16 (47.1)	
III C	3 (5.5)	8 (23.5)	
PLR > median			
II–III A	28 (59.6)	15 (35.7)	**<0.001**
III B	17 (36.2)	11 (26.2)	
III C	2 (4.3)	16 (38.1)	

Bold font indicates statistical significance. EMVI, extramural vascular invasion; LMR, lymphocyte-to-monocyte ratio; NLR, neutrophil-to-monocyte ratio; PLR, platelet-to-lymphocyte ratio. Data presented as n (% of EMVI−/ EMVI + group). Dependencies were analysed with chi-square test.

**Table 6:** Correlation analysis between CEA, CA19-9 and LMR, NLR, PLR.

Variables	CEA		CA19-9	
	tau-b	*p*	tau-b	*p*
LMR	0.03	0.634	−0.06	0.306
NLR	<0.01	0.964	0.04	0.526
PLR	0.04	0.506	−0.07	0.226

LMR, lymphocyte-to-monocyte ratio; NLR, neutrophil-to-monocyte ratio; PLR, platelet-to-lymphocyte ratio; CEA, carcinoembryonic antigen; CA19-9, carbohydrate antigen 19-9; tau-b, Kendall’s correlation coefficient; *p*, *p* value for correlation analysis.

## Data Availability

The data presented in this study are available on request from the corresponding author. The data are not publicly available due to medical data privacy issues.

## Supplementary Materials

The following supporting information can be downloaded at: https://www.mdpi.com/article/10.3390/curroncol30010043/s1, Table S1: Change between MRI-based clinical TNM-staging and post-surgical pathological TNM-staging depending on the pre-treatment status of EMVI.
