# Supplementary material for: Correlation between Lymphocyte-to-Monocyte Ratio (LMR), Neutrophil-to-Lymphocyte Ratio (NLR), Platelet-to-Lymphocyte Ratio (PLR) and Extramural Vascular Invasion (EMVI) in Locally Advanced Rectal Cancer
Source: Curr Oncol. 2022 Dec 30;30(1):545–58. doi: 10.3390/curroncol30010043 (PMC9857771; doi:10.3390/curroncol30010043)
Supplement: Supplementary file 1 [file curroncol-30-00043-s001.zip › curroncol-2108144-supplementary.pdf]

Current Oncology

Supplementals

Table S1. Change between MRI-based clinical TNM-staging and post-surgical pathological TNM-staging depending on the pre-treatment status of EMVI.

Characteristics after Surgery vs. before	EMVI−	EMVI+	*p*
Stage, n (%)			
lower	51 (56.7)	23 (38.3)	
same level	34 (37.8)	31 (51.7)	0.086
higher	5 (5.6)	6 (10.0)	
T, n (%)			
lower	43 (48.9)	26 (44.1)	
same level	44 (50.0)	31 (52.5)	0.622
higher	1 (1.1)	2 (3.4)	
N, n (%)			
lower	27 (34.2)	21 (36.2)	
same level	44 (55.7)	24 (41.4)	0.095[2]
higher	8 (10.1)	13 (22.4)	

EMVI, extramural vascular invasion. Dependencies between qualitative variables and groups were analysed with Fisher's exact test or with chi-square test[2].
